# A strategy to obtain recombinant cell lines with high expression levels. Lentiviral vector-mediated transgenesis

**DOI:** 10.1186/1753-6561-5-S8-P7

**Published:** 2011-11-22

**Authors:** Claudio Prieto, Diego Fontana, Marina Etcheverrigaray, Ricardo Kratje

**Affiliations:** 1 Cell Culture Laboratory, School of Biochemistry and Biological Sciences, Universidad Nacional del Litoral. Ciudad Universitaria – C.C.242 – (S3000ZAA) Santa Fe, Provincia de Santa Fe, Argentina

## Background

The primary goal of any recombinant protein production is to achieve successful gene transfer and expression in a target cell. There are two general categories of delivery vehicles/vectors employed in protein expression protocols. The first category includes the non-viral vectors, ranging from direct injection of DNA to complexing DNA with cationc lipds, polylysine, etc. The second category comprises DNA and RNA viral vectors.

Viruses have evolved specific mechanism to deliver their genetic material to target cell nuclei. Virus members of family *Retroviridae*, e.g. retroviruses and lentiviruses, are among the most widely used viral vectors. The use of lentiviral vectors has been increasing because the vector system has attractive features. Lentiviruses have an advantage over retroviruses in that they can infect both dividing and non-dividing cells and therefore have attracted much attention regarding the potential as vectors for gene delivery/therapy. Once integrated into the genome, recombinant cell lines are selected using different selection mechanisms.

## Results

Lentivirus particles were produced by simultaneous co-transfection of HEK 293T cells with four plasmids. The packaging construct (pMDLg/pRRE) [1], the VSV-G-expressing construct (pMD.G) [2], the Rev-expressing construct (pRSV-Rev) [1], and the self-inactivating (SIN) lentiviral vector construct containing the green fluorescent protein (GFP) reporter gene (pLV-PLK-eGFP). The medium containing lentiviral particles was collected 48 h after transfection, clarified by centrifugation 10 min at 2000 rpm and then stored at -80°C. To determine viral titers, HEK 293T cells were seeded at 3 x 10^4^ cell/ml in 6-well plates and mantained for 18 h. The supernatant was replaced with 1 ml of diluted lentiviral particles supernatant containing pLV-PLK-GFP, followed by incubation overnight. Then, the supernatants were replaced with fresh medium. The cells were analized by flow cytometry and the percentage of GFP positive cells were counted 96 h post transduction. Titer was calculated from the dilutions at which the percentage of eGFP-positive cells fall within the range of 1-30% using the following formula [3,4]: Titer (TU/ml) = [F x C/V] x D; where TU/ml: transduction units/ml, F: frequency of GFP-positive cells, C: total number of cells in the well at the time of transduction, V: volume of inoculum in ml, and D: lentivirus dilution.

The viral titer was as high as 4.4 x 10^8^ TU/ml.

After that, HEK 293T cells were seeded at a density of 6 x 10^4^ cell/ml in 6-well plates and after 24 h the supernatant was replaced by 1 ml medium containing lentiviral vectors. 96 h post-transduction the cells were analyzed by flow cytometry (t=0); then, the cells were incubated with the puromycin selection agent to obtain stable recombinant cell lines. Two protocols were employed: A-) Single-step selection protocol: the cells were incubated with 1, 5, 10, 50, 100, 150, 200 and 250 µg/ml of puromycin in different plates, and B-) Multistep gradual selection protocol: the cells were incubated from 1 up to 250 µg/ml of puromycin, but the selection agent was gradually changed each 7 days on the same plates (Table 1).

**Table 1:** eGFP expression level of different recombinant cell lines according to puromycin concentration.

Recombinant cell line	Puromycyn (µg/ml)	Single-step selection protocol	Multistep gradual selection protocol
		Fold increase eGFP expression (X-mean)	
	
CL1	1	1.0	1.0
CL5	5	2.0	2.0
CL10	10	2.0	2.0
CL50	50	2.0	2.0
CL100	100	nv	4.5
CL150	150	nv	4.5
CL200	200	nv	6.0
CL250	250	nv	nv

nv: non viable cells

**Figure 1:**
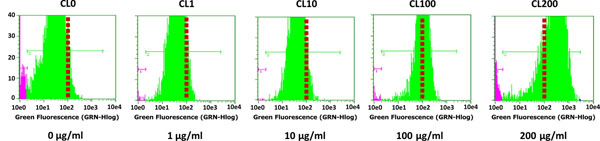
Flow cytometry of different eGFP recombinant cell lines

Once the cells were resistant to different concentrations of puromycin, recombinant cell lines were cryopreserved and analyzed by flow cytometry to compare the expression of eGFP (x-mean). Recombinant cell lines showed different eGFP expression levels according to puromycin concentration. The cell line CL200 showed the highest eGFP expression level (Figure 1).

## Conclusions

Employing the gradual selection protocol, it was possible to maintain the cells in culture condition up to 200 µg/ml puromycin and achieve higher expression levels of the reporter gene, between 2 and 6 times depending on puromycin concentration. Contrarily, in the single-step selection protocol cells cultures were resistant only up to 50 µg/ml and expression levels of eGFP were lower. Simultaneously, resistant cell lines were cloned by limit dilution methods and the resulting cell clones were also analyzed by flow cytometry. The eGFP expression of each clone was consistent with the ones observed in the respective resistant cell lines (data not shown). Therefore, with this strategy of recombinant cell line selection, it was possible to obtain high eGFP producing stable cell clones without the use of any gene amplification system.
